# Inner ear enhancement on gadolinium-enhanced 3D FLAIR images in a patient with Vogt–Koyanagi–Harada disease

**DOI:** 10.1259/bjrcr.20160090

**Published:** 2016-09-16

**Authors:** Kosuke Hida, Koichi Takano, Kengo Yoshimitsu, Jiro Fukae, Koichi Hokao

**Affiliations:** ^1^Department of Radiology, Fukuoka University Faculty of Medicine, Fukuoka, Japan; ^2^Department of Neurology, Fukuoka University Faculty of Medicine, Fukuoka, Japan; ^3^Department of Ophthalmology, Fukuoka University Faculty of Medicine, Fukuoka, Japan

## Abstract

Vogt–Koyanagi–Harada (VKH) disease is an autoimmune disorder that occurs in the melanocytes present in the uvea, leptomeninges, skin and inner ear. Clinically, this disease is characterized by bilateral uveitis and retinal detachment and is associated with meningismus and hearing loss. Gadolinium (Gd)-enhanced MRI may aid in demonstrating bilateral choroidal thickening and central nervous system involvement. We present a case of VKH where Gd-enhanced three-dimensional (3D) fluid attenuation inversion recovery (FLAIR) imaging showed abnormal bilateral enhancement in the inner ears. A 36-year-old female was referred to our institution with symptoms of visual disturbance, headache and tinnitus, and was diagnosed with VKH based on fundus examination and clinical presentations. MRI findings revealed bilateral enhancement in the choroid, leptomeninges, and inner ears. In particular, Gd-enhanced 3D FLAIR showed more conspicuous enhancement of the leptomeninges and inner ear compared with Gd-enhanced 3D *T*_1_ weighted image. Therefore, Gd-enhanced 3D FLAIR imaging can be used when leptomeningeal or inner ear pathology is clinically suspected.

## Introduction

Vogt–Koyanagi–Harada (VKH) disease is one of the causes of uveo-meningeal syndromes and is an autoimmune disease of the melanocytes present in the uvea, leptomeninges, skin, and inner ear.^1^ The revised diagnostic criteria of VKH, which was established at an international workshop on VKH, is based on clinical findings and ancillary ophthalmic tests.^2^ Although MRI findings are not included in the criteria, several studies have suggested that MRI can aid in diagnosing VKH.^3,4^ On MRI, in addition to the bilateral ocular findings, central nervous system (CNS) involvement, such as focal brain parenchymal, pachymeningeal and leptomeningeal lesions, can also be demonstrated. We present a case of VKH in which gadolinium (Gd)-enhanced three-dimensional (3D) fluid attenuation inversion recovery (FLAIR) showed abnormal bilateral enhancement in the choroid, leptomeninges, and inner ears. To the best of our knowledge, there is only one report that described inner ear involvement on MRI,^5^ and no report on inner ear enhancement on Gd-enhanced 3D FLAIR imaging.

## Case report

**Figure 1.**
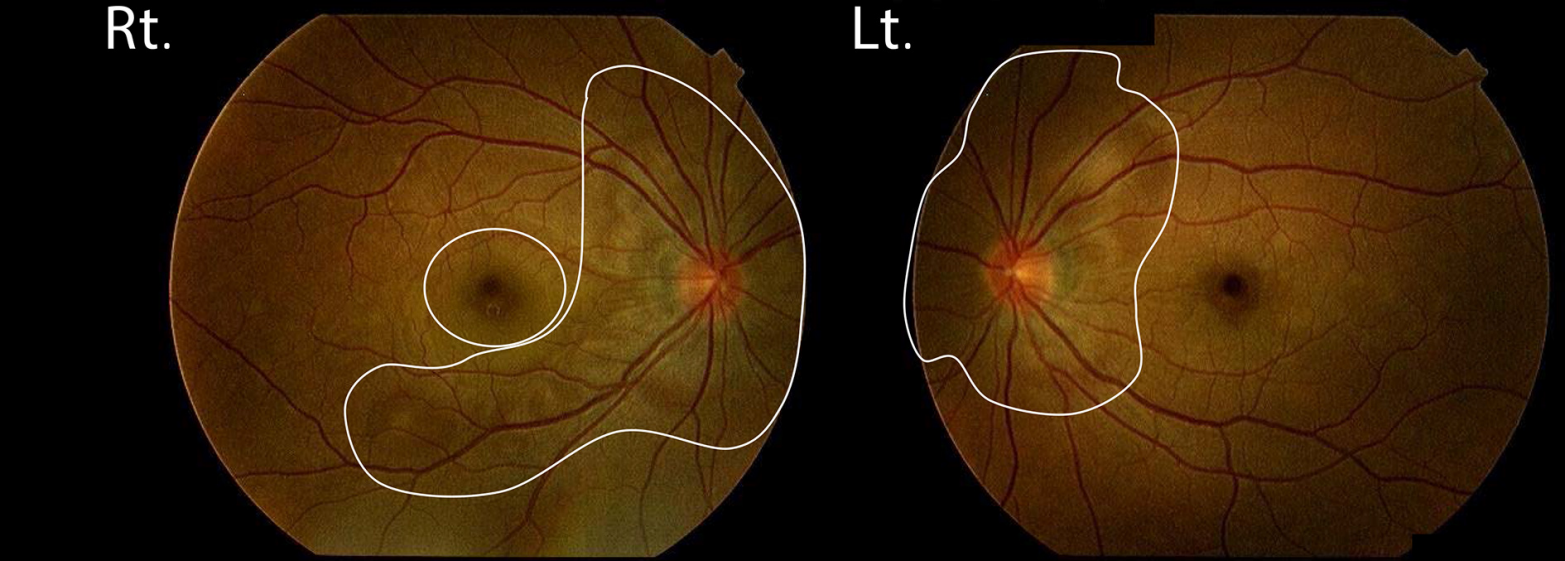
Fundus photography reveals serous retinal detachment (white lines in Rt. and Lt.) in the posterior pole of both eyes.

A 36-year-old Japanese female presented at a local hospital with a 6-day history of throbbing headache, tinnitus and blurred vision. The next day, she was referred to our hospital with positive meningeal signs and cerebrospinal fluid (CSF) lymphocytic pleocytosis. Her past medical history was unremarkable, and she had no history of ocular trauma or surgery. Her vital signs were normal, and laboratory findings were unremarkable, except for the CSF test (Table 1). Goldmann perimeter showed a central scotoma in the right eye. Fundus photography revealed serous retinal detachment in the posterior pole of both eyes (Figure 1). Optical coherence tomography demonstrated subretinal oedema and macular detachment in the right eye. Auditory examination revealed no hearing loss. There were no dermatological findings, such as poliosis, alopecia, and cutaneous vitiligo. VKH was diagnosed based on the presence of meningeal irritation, serous retinal detachment, and lack of past history of ocular trauma or surgery.

**Table 1.** Laboratory data

Complete blood count and biochemical test		Immunological test	
White blood cells	4700 µl	Antinuclear antibody (enzyme-linked immunosorbent assay)	(–)
Red blood cells	409 × 10^4^ μl^–1^	Anti Sjögren syndrome type A/B	<7.0 / <7.0
Haemoglobin	11.9 g dl^–1^	Rheumatoid factor	6
Haematocrit	35.1%	Cerebrospinal fluid test	
Platelets	20.1 × 10^4^ μl^–1^	Cells (≤5 μl^–1^)	315 μl^–1^
C-reactive protein	0.01 mg dl^–1^	Lymphocytes	98%
Creatinine	0.56 mg dl^–1^	Monocytes	2%
Total bilirubin	1.1 mg dl^–1^	Immunoglobulin G (870–1700 mg dl^–1^)	1054 mg dl^–1^
Alanine aminotransferase/aspartate aminotransferase	14/9 U l^–1^	Protein (8–43 mg dl^–1^)	172 mg dl^–1^
Uric acid	1.4 mg dl^–1^	Glucose (50–75 mg dl^–1^)	55 mg dl^–1^
Soluble interleukin 2 receptor	<100 U ml^–1^		

## Radiological findings

**Figure 2.**
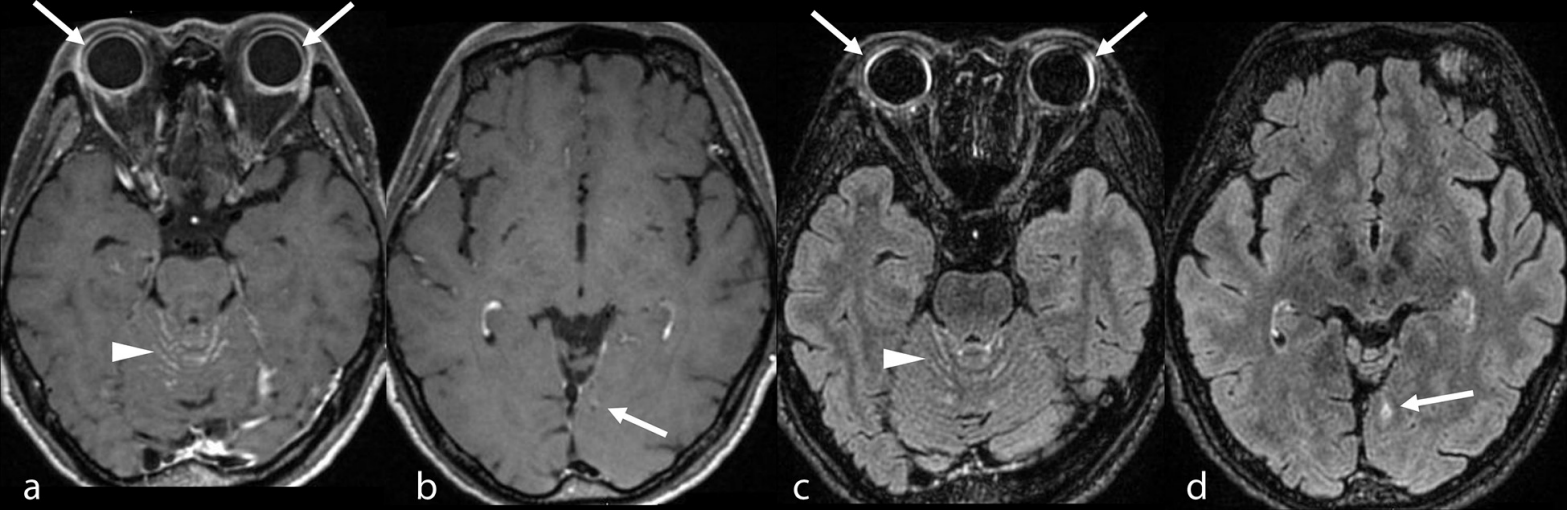
Central nervous system findings on MRI. Gd-enhanced 3D *T*_1_ weighted image shows abnormal enhancement in the bilateral choroid (arrows in a), bilateral cerebellar fissures (arrowhead in a) and leptomeninges of the left occipital lobe (arrow in b). Gd-enhanced 3D fluid attenuation inversion recovery images reveal a more conspicuous enhancement in the bilateral choroid (arrows in c) and leptomeninges of the left occipital lobes (arrow in d) compared with Gd-enhanced 3D *T*_1_ weighted image, although cerebellar fissure enhancement is similar to Gd-enhanced 3D *T*_1_ weighted image (arrowhead in c). Gd, gadolinium; 3D, three-dimensional.

**Figure 3.**
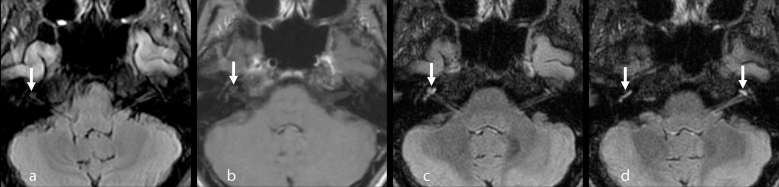
Inner ear findings on MRI. A faintly increased signal is suspected in the right cochlea on two-dimensional FLAIR image (arrow in a). There are no abnormal findings in the left cochlea. Gd-enhanced 3D *T*_1_ weighted image shows a faint enhancement in the right middle turn of the cochlea (arrow in b). Gd-enhanced 3D FLAIR images shows more conspicuous enhancement in the right apical to basal turn of the cochlea (arrow in c and right arrow in d) and left apical turn of the cochlea (left arrow in d) compared with Gd-enhanced 3D *T*_1_ weighted image (3D FLAIR images are multiplanar reconstruction images, with the location and slice thickness adjusted to two-dimensional FLAIR). FLAIR, fluid attenuation inversion recovery; 3D, three-dimensional.

Four days after admission, a brain MRI was performed using a 3.0 T scanner (Discovery 750 W, GE, Milwaukee, WI, USA) to eliminate the possibility of intracranial abnormalities. The sequences included pre-contrast two-dimensional (2D) FLAIR, pre- and post-contrast 3D *T*_1_ weighted images (CUBE *T*_1_ weighted image), and post-contrast 3D FLAIR (CUBE FLAIR). CUBE is a single slab fast-spin echo based on 3D sequence for isotropic resolution. Fat suppression was applied to all sequences. 2D FLAIR images showed increased signal intensity in the bilateral choroid. Gd-enhanced 3D *T*_1_ weighted image showed abnormal enhancement in the bilateral choroid, bilateral cerebellar fissure, and leptomeninges of the left occipital lobe. Gd-enhanced 3D FLAIR revealed more conspicuous enhancement in the bilateral choroid and leptomeninges of the left occipital lobe compared with Gd-enhanced 3D *T*_1_ weighted image, although cerebellar fissure enhancement was similar to that in Gd-enhanced 3D *T*_1_ weighted image (Figure 2). In addition, a faintly increased signal intensity in the right cochlea was suspected on 2D FLAIR. Gd-enhanced 3D *T*_1_ weighted image showed a faint enhancement in the right cochlea, and Gd-enhanced 3D FLAIR showed conspicuous bilateral enhancement in the cochlea (Figure 3).

## Treatment and outcome

From the day of admission, the patient received high-dose intravenous methylprednisolone (1000 mg day^–1^) for 3 days, followed by oral prednisolone (40 mg day^–1^). Her symptoms gradually improved and the prednisolone was tapered off. She was discharged 26 days after admission. At 1 month after discharge, her visual acuity improved and the headache did not recur.

## Discussion

VKH is a systemic inflammatory disorder characterized by bilateral uveitis associated with serous retinal detachment and accompanied by neurologic, auditory, and integumentary manifestations. Although the pathophysiology of VKH remains unknown, it is thought to be a T-cell-mediated autoimmune reaction against melanocytes.^1^ This hypothesis could explain why VKH affects melanocyte-rich tissues, such as the choroid, skin, inner ear and leptomeninges.

VKH is more prevalent in people with darker skin pigmentation, such as Asian Indians, American Indians, Mexican Mestizos and individuals from the Middle East. VKH predominates in patients aged between 20 and 50 years, with a 2 : 1 female predilection.^1^ The common symptoms are headaches (49%), photophobia (48%), tinnitus (36%), neck stiffness (33%) and hearing difficulty (32%).^6^

According to the development of imaging technology, VKH involvement demonstrated on MRI has been reported recently.^3-5, 7-9^ In addition to the ocular findings, several CNS lesions were also described. Lohman et al^3^ reported multifocal leptomeningeal enhancement within the interpeduncular fossa, cerebellar folia, and cerebral sulci on post-contrast FLAIR, which demonstrated more conspicuous enhancement than *T*_1_ weighted image. They described that the leptomeningeal enhancement in VKH may be a marker of early CNS involvement.^3^ Pachymeningeal enhancement was reported by Han et al,^4^ and Sheriff et al^7^ also described dural thickening and cerebellopontine angle mass in addition to the pachymeningeal enhancement.^8^ These findings suggest that melanocyte-rich organs were affected by VKH, although several studies have also reported parenchymal brain lesions.^7,9^

In the present case, we obtained Gd-enhanced 3D FLAIR after Gd-enhanced 3D *T*_1_ weighted image. We routinely included Gd-enhanced 3D FLAIR sequence in brain MRI when pia–arachnoid involvement was suspected, because Gd-enhanced 3D FLAIR often demonstrates conspicuous superficial enhancement, as observed in this case. Fukuoka et al^10^ reported that Gd-enhanced 3D FLAIR provided more information than Gd-enhanced 3D *T*_1_ weighted images for the depiction of leptomeningeal diseases. Gd-enhanced 3D FLAIR may be superior to *T*_1_ weighted imaging in detecting abnormal leptomeningeal enhancement owing to the lack of enhancement in the vasculatures at normal flow,^10^ effective suppression of CSF flow artefacts^11^ and higher sensitivity at low Gd concentrations.^10–12^

In our case, Gd-enhanced 3D FLAIR showed more conspicuous enhancement than Gd-enhanced 3D *T*_1_ weighted image in the leptomeninges and bilateral inner ears. To our knowledge, there is only one report, which describes inner ear involvement on MRI in a patient with VKH, although the details of the imaging sequences were not provided.^5^ Abnormal enhancement of the cochlea has been reported in inflammatory diseases such as bacterial meningitis, acute otitis media, and Wegener granulomatosis.^13^ As VKH patients have a high incidence of cochlear and vestibular involvement,^14^ abnormal Gd-enhanced FLAIR enhancement in our case may represent inner ear inflammation associated with VKH. A recent article suggested that intravenous Gd injection is useful for evaluating the blood–labyrinth barrier in patients with inner ear diseases on 3D FLAIR.^12^ Yoshida et al^15^ reported that patients with sudden sensorineural hearing loss demonstrated enhancement in the inner ear on Gd-enhanced 3D FLAIR, although no enhancement was observed on Gd-enhanced 3D *T*_1_ weighted image.^15^ In our case, abnormal enhancement was only faintly detected on Gd-enhanced 3D *T*_1_ weighted image in the inner ear. Therefore, Gd-enhanced 3D FLAIR may be more sensitive than Gd-enhanced 3D *T*_1_ weighted image in detecting inner ear pathology. Although we did not obtain pre-contrast 3D FLAIR images, multiplanar reconstruction images created from the Gd-enhanced 3D FLAIR, with the location and slice thickness adjusted to that of 2D FLAIR, revealed an obviously increased signal in the inner ear compared to the 2D FLAIR (Figure 3). Therefore, we can assume that the findings on Gd-enhanced 3D FLAIR in our case represent abnormal enhancement, which may reflect an inflammatory process in the inner ear.

## Conclusions

MRI can be used to detect ocular and CNS diseases, and can also aid in the detection of inner ear involvement in patients with VKH. Gd-enhanced 3D FLAIR is recommended when leptomeningeal or inner ear pathology is clinically suspected.

## Learning points

1. On MRI, in addition to the bilateral ocular findings, CNS involvement such as focal brain parenchymal, pachymeningeal and leptomeningeal lesions, may be also demonstrated in patients with VKH.
2. Gd-enhanced MRI may represent inner ear inflammation associated with VKH.
3. Gd-enhanced 3D FLAIR may be more sensitive than Gd-enhanced 3D *T*_1_ weighted image in detecting leptomeningeal and inner ear pathology.

## Consent

Written informed consent for the case to be published (including images, case history and data) was obtained from the patient for publication of this case report.
